# Inactivation of human plasma alters the structure and biomechanical properties of engineered tissues

**DOI:** 10.3389/fbioe.2022.908250

**Published:** 2022-08-23

**Authors:** Cristina Rosell-Valle, María Martín-López, Fernando Campos, Jesús Chato-Astrain, Rafael Campos-Cuerva, Miguel Alaminos, Mónica Santos González

**Affiliations:** ^1^ Unidad de Producción y Reprogramación Celular de Sevilla (UPRC), Red Andaluza de Diseño y Traslación de Terapias Avanzadas (RADyTTA), Seville, Spain; ^2^ Escuela Internacional de Doctorado Universidad de Sevilla, Seville, Spain; ^3^ Tissue Engineering Group, Department of Histology, Universidad de Granada, Granada, Spain; ^4^ Instituto de Investigación Biosanitaria ibs. Granada, Granada, Spain; ^5^ Centro de Transfusiones, Tejidos y Células de Sevilla (CTTS), Fundación Pública Andaluza para la Gestión de la Investigación en Salud en Sevilla (FISEVI), Seville, Spain

**Keywords:** fibrin-agarose hydrogel, tissue engineering, pathogen reduction method, biomechanical properties, bioartificial skin

## Abstract

Fibrin is widely used for tissue engineering applications. The use of blood derivatives, however, carries a high risk of transmission of infectious agents, necessitating the application of pathogen reduction technology (PRT). The impact of this process on the structural and biomechanical properties of the final products is unknown. We used normal plasma (PLc) and plasma inactivated by riboflavin and ultraviolet light exposure (PLi) to manufacture nanostructured cellularized fibrin-agarose hydrogels (NFAHs), and then compared their structural and biomechanical properties. We also measured functional protein C, prothrombin time (PT), activated partial thromboplastin time (APTT), thrombin time (TT) and coagulation factors [fibrinogen, Factor (F) V, FVIII, FX, FXI, FXIII] in plasma samples before and after inactivation. The use of PLi to manufacture cellularized NFAHs increased the interfibrillar spacing and modified their biomechanical properties as compared with cellularized NFAH manufactured with PLc. PLi was also associated with a significant reduction in functional protein C, FV, FX, and FXI, and an increase in the international normalized ratio (derived from the PT), APTT, and TT. Our findings demonstrate that the use of PRT for fibrin-agarose bioartificial tissue manufacturing does not adequately preserve the structural and biomechanical properties of the product. Further investigations into PRT-induced changes are warranted to determine the applications of NFAH manufactured with inactivated plasma as a medicinal product.

## Introduction

Tissue engineering is an emerging technology in regenerative medicine that combines cells, biomaterials and biochemical and physicochemical factors to restore tissue and organ function (Bakhshandeh et al., 2018; Kwon et al., 2018). Several types of scaffolds and nanoparticles have been developed based on the extracellular matrix of human tissues, to improve their biological, chemical and mechanical properties (Hinderer et al., 2016; Hasan et al., 2018). Numerous synthetic and natural polymers are currently available to produce biomaterials, which are defined as substances engineered to functionally interact with biological systems. A good example of this is the biopolymer fibrin, which is widely used for tissue engineering applications as both a medical device and a biomaterial itself (García Delgado et al., 2019; de Melo et al., 2020; Arai et al., 2021). Fibrin is generated from fibrinogen and is involved in essential biological functions such as wound repair by providing a temporary matrix to support cell growth, invasion, attachment and biochemical activity (Kearney et al., 2021). The US Food and Drug Administration has approved several fibrin-based products as hemostatic sealants and adhesives (Spotnitz, 2014). Fibrin can be combined with agarose in the fabrication of bioartificial tissues, since this combination of fibrin and agarose demonstrated to provide the tissue with adequate biomechanical properties, including the Young modulus, stress at fracture and traction deformation, and permits its use as scaffolds (Ionescu et al., 2020). Several preclinical studies have successfully demonstrated the application of fibrin-agarose hydrogels in the repair of damaged human organs such as the cornea (Alaminos et al., 2006), skin (Carriel et al., 2012), nerves (Chato-Astrain et al., 2018), oral mucosa (Fernández-Valadés-Gámez et al., 2016) and cartilage (García-Martínez et al., 2017). These positive results have paved the way for the clinical translation of two bioartificial tissues, which are currently used for severe skin burns (Egea-Guerrero et al., 2019) and corneal ulcers (Rico-Sánchez et al., 2019).

Human fibrin is a component of blood plasma. While blood components have had a positive impact on the manufacture of advanced therapy medicinal products (Allain et al., 2005), the use of these raw materials carries a risk of pathogen transmission to patients (Klein et al., 2007; Lanteri et al., 2016; Motta et al., 2016). For this reason, regulatory agencies have issued guidelines to prevent and control the transmission of infectious agents [European Pharmacopoeia, 2013; European Directorate for the Quality of Medicines & HealthCare (EDQM), 2017]. In recent years, a number of pathogen reduction technology (PRT) systems have been developed to minimize pathogens in plasma and platelets (Reikvam et al., 2010; Kaiser-Guignard et al., 2014; Viau et al., 2017). The most commonly used PRT is the Mirasol™ (PRT) System (Marschner and Goodrich, 2011; Kwon et al., 2014), which is based on photochemical principles to cross-link the nucleic acids of pathogens. The method uses riboflavin (vitamin B2), which binds to nucleic acids after ultraviolet A (UVA) light exposure, causing the oxidation of proteins and irreversible damage by blocking replication of pathogen DNA (Kumar et al., 2004). Although PRT is desirable and beneficial, whether riboflavin and UVA exposure affects blood derivatives used to manufacture bioartificial tissues has not been tested. Various studies report that PRT can alter the metabolic (pH, sugars, nucleosides) and physiological parameters of platelets (Janetzko et al., 2005; Kerkhoffs et al., 2010; Stivala et al., 2017) and lead to direct modifications of fibrinogen (Hubbard et al., 2016).

Here, we used human non-inactivated plasma (PLc) or plasma inactivated with riboflavin/UVA light exposure (PLi) to fabricate bioartificial tissues based on fibrin-agarose hydrogels. To better understand the effect of PLi on the generation of the hydrogels, we analyzed their structure and biomechanical properties. In addition, we evaluated the effect of riboflavin/UVA exposure on the levels of natural anticoagulants and coagulation factors present in plasma and questioned whether this contributed to modify the essential properties of bioartificial tissues.

## Material and methods

### Pathogen reduction method

**FIGURE 1:**
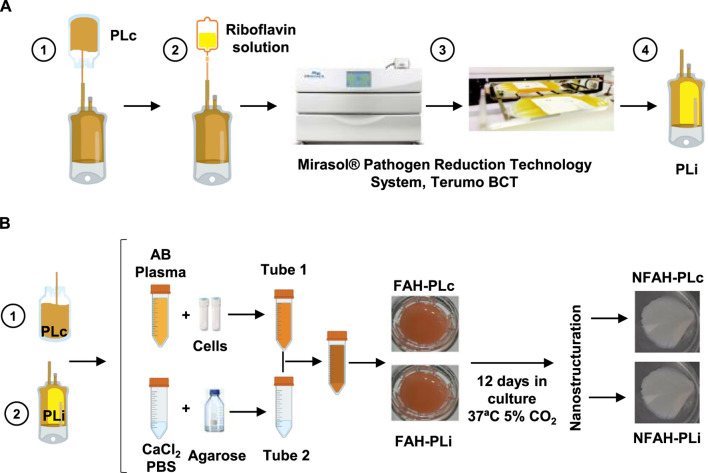
Inactivation of human plasma and generation of the cellularized nanostructured fibrin-agarose hydrogel. **(A)** Schematic representation of human plasma treated with the Mirasol^®^ Pathogen Reduction Technology System. **(B)** Experimental design of the generation of nanostructured fibrin-agarose hydrogel with embedded allogeneic dermal fibroblasts with PLc and PLi. PLc, non-inactivated plasma; PLi, inactivated plasma; CaCl_2_, calcium chloride; PBS, phosphate buffered saline; FAH, fibrin-agarose hydrogel; NFAH, nanostructured fibrin-agarose hydrogel.

Three plasmapheresis units obtained from AB blood-type donors were treated in the Mirasol™ (PRT) System (Terumo BCT, Zaventem, Belgium), as described (Larrea et al., 2009; Smith and Rock, 2010) (Figure 1A). Group AB plasma was used to prevent potential problems with antigens and isoagglutinins Briefly, 273 ml of plasma was transferred to an illumination bag containing 35 ± 5 ml of a sterile riboflavin solution (500 μM) (Terumo BCT). The bag was placed in the illuminator for UVA light exposure, with a linear agitation of 120 cpm at room temperature (RT). The PLi was then transferred to a storage bag and frozen simultaneously (−80°C) with its matched control (PLc) product.

### Generation of cellularized nanostructured fibrin-agarose hydrogels

Nanostructured fibrin-agarose hydrogels (NFAHs) cellularized with human dermal fibroblasts were generated with PLc (NFAH PLc) or PLi (NFAH PLi) (Figure 1B) by described protocols (Fernández-Valadés-Gámez et al., 2016; García-Martínez et al., 2017; Ionescu et al., 2020). In brief, 25 ml of AB-type human plasma (PLi or PLc) was mixed in one tube (Tube 1) with Dulbecco’s modified Eagle’s medium (DMEM) supplemented with 10% fetal bovine serum and 0.5 ml of tranexamic acid (Rottapharm, Milan, Italy) and containing dermal fibroblasts (150,000 cells/mL). The mixture was combined with 1.8 ml of 10% calcium chloride (B. Braun, Melsungen, Germany) and 1.5 ml of liquid 2.2% type VII agarose diluted in phosphate buffered saline (PBS; Sigma-Aldrich, St. Louis, MO, United States), previously prepared in a second tube (Tube 2). The mixture was rapidly aliquoted into a T6 multi-well plate (Corning Gosselin S.A.S. Life Sciences, Hazebrouk Cedex France) and allowed to solidify at 37°C. Six samples of each type (NFAH PLc and NFAH PLi) were generated. The hydrogels were then covered with DMEM containing 1% non-essential amino acids (100×) (Sigma-Aldrich), 20 μg/ml gentamycin (Normon, Madrid, Spain), 1% GlutaMax™ (Life Technologies, Madrid, Spain), 5% human platelet lysate solution (Fernández Muñoz et al., 2021), and stored for 12 days at 37°C/5% CO_2_ prior to the nanostructuring process (Campos-Cuerva et al., 2019).

### Cell viability analysis in cellularized nanostructured fibrin-agarose hydrogels

Cell viability was determined with the LIVE/DEAD^®^ Viability/Cytotoxicity Kit for mammalian cells (Molecular Probes/Invitrogen, Thermo Fisher Scientific, Waltham, MA, United States) immediately after the nanostructuring process. NFAH samples were washed three times with PBS. The samples were then incubated with the staining solution for 30 min at RT and washed three times with PBS. The number of live and dead cells was determined by fluorescence microscopy, and the cell viability (percentage) was calculated for each study group. Three counts were made for each group of manufactured NFAH.

### Structural analysis in cellularized nanostructured fibrin-agarose hydrogels

NFAH samples were fixed in 3.7% buffered formaldehyde at RT, then dehydrated and embedded in paraffin following a standard tissue-processing protocol. NFAH sections were stained with hematoxylin and eosin and examined using a Nikon Eclipse 90i light microscope (Serrato et al., 2009). The porosity of each type of NFAH was determined by quantifying the interfibrillar spaces in the fibrin-agarose fibrillary mesh using ImageJ software (Schneider et al., 2012). Six analyses were made for each group of manufactured NFAH.

### Biomechanical analysis of cellularized nanostructured fibrin-agarose hydrogels

The biomechanical properties of the samples (cellularized NFAH PLc and NFAH PLi) were evaluated immediately after nanostructuring (Ionescu et al., 2020). All samples were subjected to tensile testing using an electromechanical material testing instrument (Instron, Model 3345-K3327). Samples were first sectioned to a regular rectangular shape, oriented with their major length along the direction of tension, and clamped at each end. A constant distance of 1 cm between the clamps and the sample was maintained. Analyses were run at a constant strain rate of 5 mm per min at RT to assess the following parameters: Young’s modulus (calculated as the tangent modulus of the initial, linear portion of the stress–strain curve of each experimental run), stress at fracture break, and traction deformation. A 50-N Instron load cell was used to obtain the data for the stress–strain curves.

### Protein C, prothrombin time, activated partial thromboplastin time and thrombin time assays

Functional protein C, prothrombin time (PT), activated partial thromboplastin time (APTT) and thrombin time (TT) were measured by clotting assays (STA NeoPTimal, STA Cephascreen and STA Thrombin kits, respectively; Diagnostica Stago S.A.S., Asnières sur Seine, France). The international normalized ratio (INR) was derived from the PT and was calculated as the ratio of the PT of the PLi sample to a control PLc PT using the following formula: INR = PLi PT/PLc PT (Shikdar et al., 2021). Samples were analyzed at Echevarne Laboratories, Seville, Spain.

### Factor V, VIII, X, XI, XIII and fibrinogen analysis

Factor (F) V, FVIII, FX, FXI, FXIII and fibrinogen were measured by clotting assays (STA Deficient V, STA InmunoDef VIII, STA C.K.Prest, STA InmunoDef XI, K-Assay Factor XIII and STA Liquid Fib Kits Diagnostica Stago S.A.S., respectively). Measurements detect factor activity, excepting factor XIII and fibrinogen assays, which detect factor levels. Samples were analyzed at Echevarne Laboratories, Seville, Spain.

### Statistical analysis

Results are presented as mean ± standard error of the mean (SEM). The Shapiro–Wilk test was used to assess normal distribution of data. A two-tailed unpaired Student’s t-test was used to compare the structural and biomechanical properties. To identify statistical differences between samples corresponding to NFAH PLc and NFAH PLi, we performed a paired Student’s t-test. Correlation analysis was performed using the Pearson correlation coefficient (value ranges from -1 to +1). *p*-values lower than or equal to 0.05 were considered as statistically significant. All statistical analyses were performed with GraphPad Prism, version 8.0. (GraphPad Software Inc., San Diego, CA).

## Results

### Structural and biomechanical properties of bioartificial tissues manufactured with PLi or PLc

**FIGURE 2:**
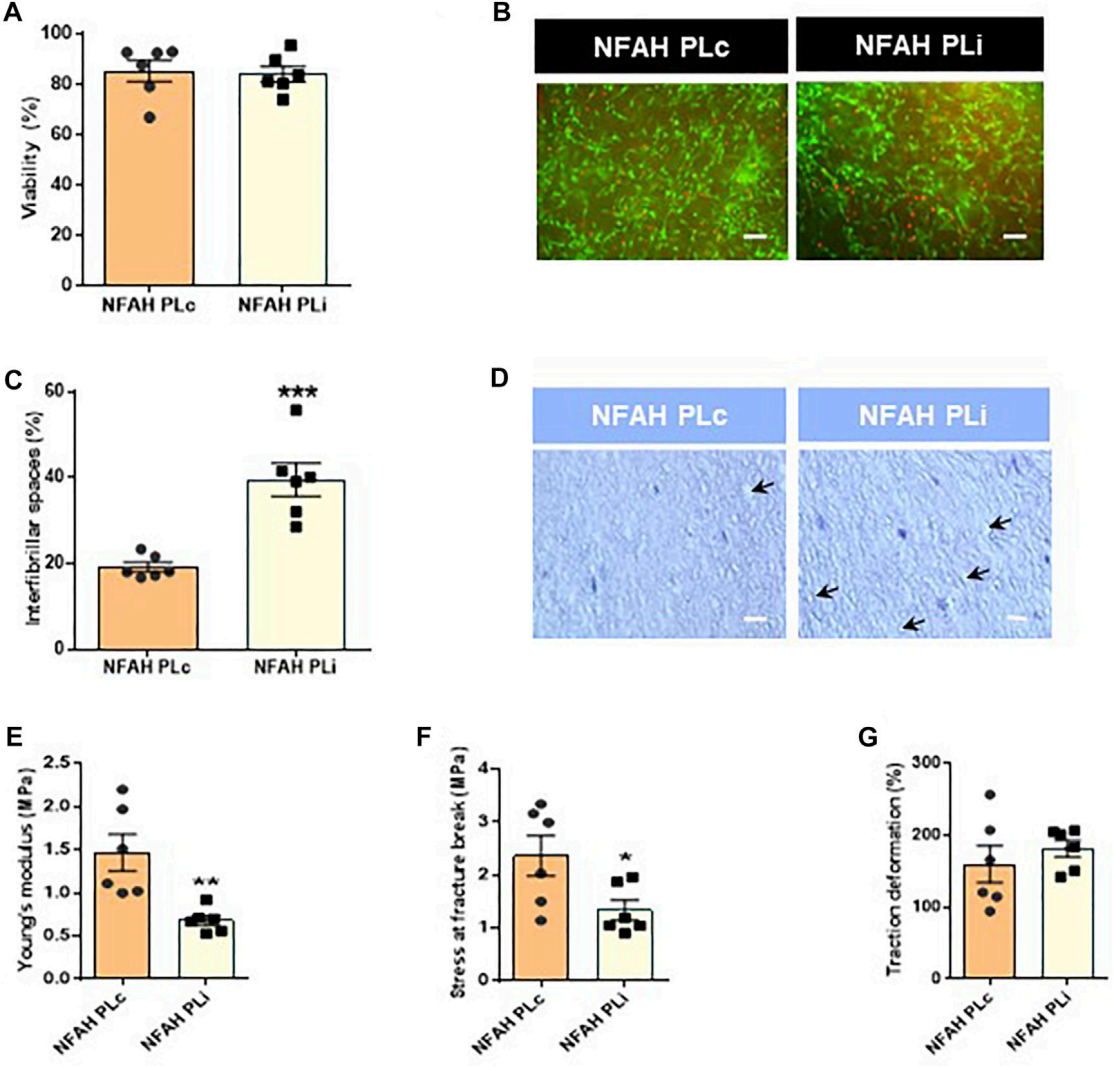
Structural and biomechanical characterization of nanostructured fibrin-agarose hydrogels. Bar graph representation of **(A)** percentage of cell viability and **(B)** LIVE/DEAD^®^ Assay staining of cellular NFAH (scale bar: 100 μm). **(C)** Interfibrillar spacing immediately after the nanostructuring process. **(D)** Representative hematoxylin/eosin staining of tissue sections of cellular NFAHs (scale bar: 100 μm). Biomechanical analysis: Bar graph representation of **(E)** Young’s modulus, **(F)** stress at fracture-break, and **(G)** traction deformation. Data are shown as mean ± SEM. Arrows indicate interfibrillar spacing. Six independent samples per group were analyzed. Two-tailed unpaired Student’s *t*-test: *p < 0.05; ***p < 0.0001.

We first questioned whether NFAHs manufactured with PLi exhibited alterations in cell viability, porosity degree and biomechanical properties. No significant differences were observed between NFAH PLi and NFAH PLc in cell viability (*p* > 0.05; Figures 2A,B). By contrast, PLi negatively affected the structure of NFAH, as shown by a significantly higher interfibrillar spacing (porosity) in NFAH PLi than in NFAH PLc (t_10_ = 5.087, *p* < 0.001; Figures 2C,D), suggesting that the application of PRT resulted in a loss of some essential properties of the resulting bioartificial tissues.

Analysis of the biomechanical properties of both hydrogels revealed other significant differences. Specifically, we found significantly lower values for Young’s modulus and stress at fracture break in NFAH PLi as compared with NFAH PLc (t_10_ = 3.599, *p* < 0.05; t_10_ = 2.418, *p* < 0.05, respectively, Figures 2E,F). No significant differences were found between NFAH PLi and NFAH PLc for the traction deformation tests (*p* > 0.05; Figure 2G).

### Implications of pathogen reduction technology for human plasma quality

We next comparatively analyzed several factors present in human plasma to determine the putative effects of PRT on hydrogels based on human plasma. We focused on measuring factors VIII, XI and fibrinogen because they are the most affected by plasma inactivation (Larrea et al., 2009). Besides, we chose factors V, VIII, X and fibrinogen that form part of the common pathway together with factor II, prothrombin (Chaudhry and Babiker, 2018). For the later, we measured prothrombin time, PT. This test measures, as a whole, the activity of factors II, V, VII, X and fibrinogen. Similarly, we measured thromboplastin time, APTT, which explores coagulation factors XII, XI, X, VIII, X, II and fibrinogen. Additionally, we measured factor XIII, considering it as crucial for our product since it promotes fibrin crosslinking (Beckman et al., 2018).

**FIGURE 3:**
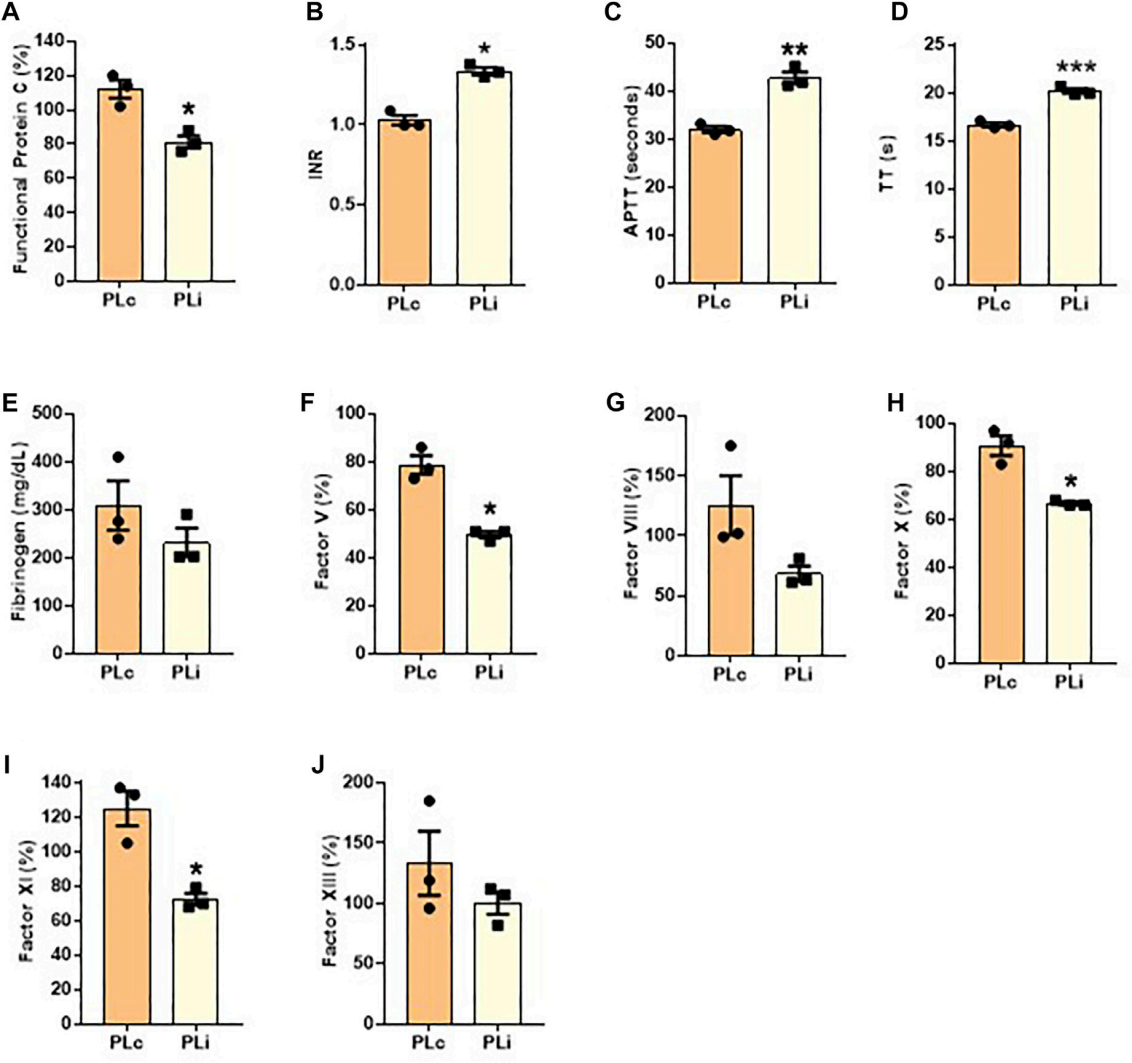
Coagulation profile of human plasma after pathogen reduction. The following measurement were performed: **(A)** functional protein C, **(B)** international normalized ratio (INR), **(C)** activated partial thromboplastin time (APTT), **(D)** thrombin time (TT), coagulation factor **(E)** V, **(F)** X, **(G)** XI, **(H)** VIII, and **(I)** XIII and **(J)** fibrinogen. Data are shown as mean ± SEM. Three independent samples per group were analyzed. Student’s paired *t*-test: *p < 0.05; **p < 0.01; ***p < 0.001.

As shown in Figure 3, we found a significant decrease in the percentage of functional protein C (t_2_ = 6.651, *p* < 0.05; Figure 3A), and an increase in INR (t_2_ = 18.38, *p* < 0.05; Figure 3B), APTT (t_2_ = 18.38, *p* < 0.05; Figure 3C) and TT (t_2_ = 6.08, *p* < 0.001; Figure 3D) in PLi as compared with PLc. In addition, the percentage of the coagulation factors FV (t_2_ = 7.666, *p* < 0.05), FX (t_2_ = 6.656, *p* < 0.05), and FXI (t_2_ = 5.812, *p* < 0.05) was significantly lower in PLi than in PLc (Figures 3E–G, respectively). PLi samples also showed a slight decrease in the levels of FVIII, FXIII and fibrinogen compared with PLc samples, but the differences were not significant (*p* > 0.05, Figures 3H–J, respectively).

### Correlation between the structural and biomechanical properties of bioartificial tissues and coagulation factor levels

To determine whether there was a relationship between the main changes found in the structural and biomechanical properties and changes in coagulation factors after the application of PRT, we performed correlation analyses (Table 1). Results of these analyses showed that the interfibrillar spacing (porosity) of the NFAH negatively correlated with FV (r = −0.87, *p* < 0.05), FX (r = −0.87, *p* < 0.05) and FXI (r = −0.85, *p* < 0.05). Regarding the biomechanical properties, we found that Young’s modulus positively correlated with FV (r = 0.84, *p* < 0.05) and FX (r = 0.79, *p* < 0.05), whereas the stress at fracture break positively correlated with FV (r = 0.83, *p* < 0.05), FVIII (r = 0.83, *p* < 0.05), FX (r = 0.82, *p* < 0.05) and FXI (r = 0.82, *p* < 0.05). No correlation was found with fibrinogen level.

**TABLE 1:** Correlation matrix between structural and biomechanical properties and coagulation factors.

	Interfibrillar spaces	Young’s modulus	Stress at fracture break
Fibrinogen	−0.49	0.42	0.61
*p*	0.32	0.17	0.20
Factor V	**−0.87**	**0.84**	**0.83**
*p*	**0.02**	**0.03**	**0.04**
Factor VIII	**−0.87**	0.65	**0.83**
*p*	**0.02**	0.16	**0.04**
Factor X	**−0.87**	**0.79**	**0.82**
*p*	**0.02**	**0.05**	**0.05**
Factor XI	**−0.85**	0.77	**0.82**
*p*	**0.03**	0.07	**0.05**
Factor XIII	−0.47	0.36	0.21
*p*	0.34	0.48	0.68

Pearson’s r coefficient. Significant correlation coefficients and the corresponding significance level (p) are given in bold.

## Discussion

Fibrin-agarose hydrogels are scaffold-based tissue engineering products that are gaining widespread interest in regenerative medicine for the treatment of tissue (Campos et al., 2020). This biomaterial must comply with the basic properties of organs and tissues to which it is applied, and provide structural support for cells and mimic the mechanical properties of native tissue. Our results show that PRT modifies the porosity degree of the final product by increasing the interfibrillar spacing of the fibrin-agarose scaffold. The three-dimensional structure of NFAH PLi was compatible with a loss of integrity when compared with NFAH PLc. The pore size of biomaterials is a critical factor affecting cell attachment and cell physiology (Vu et al., 2015), and maintaining this parameter is very important for bioartificial tissues to be functional. Interestingly, the pore size modification in NFAH PLi was not associated with a decrease in cell viability. While it has been suggested that increased interfibrillar spacing may reduce the strength and flexibility of bioartificial tissues and impair their clinical use (Shahmirzadi et al., 2013), our finding that cell viability was preserved might suggest that the pore size found in both NFAH PLi and NFAH PLc could be within a physiological range.

The biomechanical properties of the manufactured NFAH PLc were similar to those previously determined for fibrin-agarose hydrogels, whereas the properties of NFAH PLi were inferior (Campos et al., 2020; Ionescu et al., 2020). Generally, maintaining the biomechanical properties of an artificial tissue is a prerequisite for tissue-engineered products for clinical use. Whether or not this difference in NFAH PLi has a clinical effect should be determined in future studies in laboratory models. However, we hypothesize that the biomechanical modifications generated by the PRT System compromises the biological function of these artificial tissues. That being said, the structural and biochemical properties are not the only key criteria to be considered for the application of artificial tissues (Linares-Gonzalez et al., 2021). Whether the positive effects of the PRT treatment––in terms of biosafety––outweigh the negative effects of the treatment should be determined on a case-by-case basis, as some applications may not require a bioartificial tissue with strong biomechanical properties.

Because fibrin is mainly obtained from donors’ human plasma, PRT is required to lessen the risk of transmission of infectious agents [Klein et al., 2007; European Pharmacopoeia, 2013; Lanteri et al., 2016; Motta et al., 2016; European Directorate for the Quality of Medicines & HealthCare (EDQM), 2017]. Nonetheless, PRT may have adverse effects on the coagulation system, which could change the functionality of the treated plasma and impair clinical applications. In this regard, several authors have analyzed the effects of PRT on plasma components, specifically on coagulation factor levels, finding a decrease in coagulation factor activity after riboflavin/UVA exposure when compared with fresh frozen plasma (Hornsey et al., 2009; Larrea et al., 2009; Smith and Rock, 2010; Marschner and Goodrich, 2011). Nevertheless, these reports concluded that it is unlikely that this decrease has any clinical impact. By contrast, Klompas and colleagues recently demonstrated that the loss of coagulation factor activity in pathogen-reduced COVID-19 convalescent plasma had a clinical impact associated with the pathogen reduction process (Klompas et al., 2022). Moreover, in agreement with our results, they demonstrated an increase in APTT and TT, suggesting that a slight prolongation of coagulation times may occur due to the combined loss of activity of several coagulation factors (Hornsey et al., 2009; Klompas et al., 2022). In line with these studies, we also found a significant reduction of functional protein C and FV, FX, and FXI levels, and a slight decrease in FVIII, FXIII and fibrinogen after inactivation. These results might partly explain the structural and biomechanical changes evident in samples generated with PRT-treated human plasma.

Considering that riboflavin/UVA treatment can act as a cross-linking system able to improve the biomechanical rigidity of decellularized scaffolds (Xiang et al., 2017) and corneas (Aslanides et al., 2016), it might also have a positive effect on fibrin-agarose scaffolds. However, the correlation analysis revealed the opposite behavior, showing that riboflavin/UVA exposure on plasma did not preserve coagulation factor levels.

Factor V is a component of the prothrombin complex, which interacts with other coagulation proteins, including activated FX and prothrombin to increase the production of thrombin. Thrombin mediates the cleavage of fibrinogen to fibrin monomers that, upon polymerization, form a fibrin clot (Kalafatis and Mann, 1997; Göbel et al., 2018). In addition, FXI also contributes to the generation of thrombin and thus, is involved in the formation of fibrin (Meijers et al., 2000). Undoubtedly, these coagulation proteins are needed to stabilize the fibrin-agarose scaffold and to maintain the fibrillar pattern of hydrogels. The observed relatedness between coagulation factors and the structure of the NFAH also suggests that the loss of their activity could affect biomechanical properties. In this regard, Young’s modulus is associated with FV and FX, whereas the stress at fracture break is related to FVIII. Overall, these findings suggest that the loss of key coagulation factors in NFAH produced with PLi is associated with the generation of a less compact fibrillary mesh, which could impact its clinical usefulness. A balance should be reached between PRT and its applicability on artificial tissues to achieve safer and more effective tissue engineered products. Given that the PRT protocol cannot be modified, the balance would rely on the supplementation with coagulation factors that aided fibrin formation prior to NFAH jellification, for example, FV or FX, which resulted significantly affected by inactivation. FX, together with FV (cofactor) convert prothrombin into thrombin, which would further activate fibrin formation and at the same time enhance the intrinsic pathway of the coagulation cascade. Other possibilities could contemplate the addition of biomaterials that reinforced the structure of the hydrogel by reducing interfibrillar spaces. Our group has explored the addition of biomaterials like genipin, which used in concentrations of 0.1%–0.5%, improved structural and biomechanical properties of NFAH (Campos et al., 2018). A different approach could be the addition of 0.7 mg/ml chitosan-silica which has been shown to improve the mechanical stability of fibrin hydrogels with low risks of cytotoxicity (Becerra et al., 2021). Nevertheless, “*in vivo*” studies are still needed to check efficacy and security of either option.

## Limitations

Our data indicate that the application of inactivation methods based on riboflavin and UV light alters the coagulation system in plasma. This disruption leads to a loss of coagulation factor activity that may affect the structure of NFAH and consequently, its biomechanical properties. Although our data are robust, we detect some limitations that should be taken into account in future studies.

It is unknown whether the application of other pathogen inactivation methods in plasma samples can mitigate the decrease in coagulation factors activity and consequently, reduce the adverse impact on the structure and biomechanical properties of these hydrogels. Therefore, others PRTs should be tested.

Another consideration could be the incubation period of cellularized fibrin-agarose hydrogels. Fibrin scaffolds accelerate fibroblast growth and remodeling of extracellular matrix (San Matin et al., 2011) thus, if a suitable structure has not formed due to reduced coagulation factors, inter-fibrillar spaces may increase with culture time. In this regard, decreasing the culture time could better preserve the structure and biomechanical properties of cellularized NFAH. In addition, it might be appropriate to explore other alternatives to reinforce the structure and functionality of cellularized NFAH such as the application of genipin (Campos et al., 2018) or the fibrin combination with polymer materials such as polycaprolactone (PCL), that promote smaller aperture and larger fiber diameter in fibrin scaffolds (Lei et al., 2020).

Regarding the effects caused by PRT, these could be compensated by coagulation factors supplementation during hydrogels manufacture, although this step may increase manufacturing costs.

## Conclusion

No studies had analyzed the impact of PLi use on bioartificial tissue manufacturing. Considering that blood derivatives must be treated with a pathogen reduction method to prevent the transmission of infectious agents, our results show that the use of riboflavin/UVA-based PLi for the fabrication of NFAH significantly affects the structural, biochemical and biological properties of the fibrin-agarose matrix, possibly due to changes in the level/activity of several coagulation factors. Future studies should evaluate the advantages and disadvantages of PLi used in the manufacturing process of this type of bioartificial tissues and the specific applications in which these changes could be acceptable for clinical use.

## Data Availability

The raw data supporting the conclusion of this article will be made available by the authors, without undue reservation.

## Abbreviations

AB plasma, plasma group AB; APTT, activated partial thromboplastin time; DMEM, Dulbecco’s modified Eagle’s medium; INR, international normalized ratio; NFAH, nanostructured fibrin-agarose hydrogel; PLc, non-inactivated human plasma; PLi, inactivated human plasma; PRT, Pathogen reduction technology; PT, prothrombin time; RT, Room temperature; TE, Tissue engineering; TT, thrombin time; UVA, ultraviolet A.
